# Has Propranolol Rendered Sclerotherapy Obsolete for Poor Risk Alcoholic Cirrhotic Patients?

**DOI:** 10.1155/1994/45741

**Published:** 1994

**Authors:** Seigo Kitano

**Affiliations:** Department of Surgery I Oita Medical University Oita 879-55 Japan


*References*

1. Layer, P. and Grandt, D. (1993) Diagnosis of pancreatic pseudocysts. In: Beger, H. J., Büchler, M. and Malfertheiner, P. (ed), Standards in pancreatic surgery. Springer-Verlag, Heidelberg-Berlin. 520–525
2. Grace, P. A. and Williamson, R. C. N. (1993) Modern management of pancreatic pseudocysts. *Br. J. Surg.*, **80**, 573–581
3. Warshaw, A. L. and Rutledge, P. L. (1987) Cystic tumours mistaken for pancreatic pseudocysts. *Ann. Surg.*, **205**, 393–398
4. Mullins, R. J., Mallangoni, M. A. and Bergamini, T. M. *et al.* (1988) Controversies in the management of pancreatic pseudocyst. *Am. J. Surg.*, **155**, 165–172
5. Scheele, J. and Altendorf-Hofmann, A. (1990) Needle track seeding from tumor biopsy on the liver. *Hepatogastroenterol*, **37**, 335–337
6. Railey, D. J., Barkin, J. J. and Livi, J. (1991) Aspiration of cystadeno carcinoma mimicking pancreatic pseudocyst. *Pancreas*, **6**, 491–492
7. Sachs, J. R., Deren, J. J., Sohn, M. and Nausbaun, M. (1989) Mucinous cystadenoma: pitfalls of differential diagnosis. *Am. J. Gastroenterol.*, **84**, 811–816
8. Geer, R. J. and Brannan, M. F. (1993) Prognostic indicators for survival after resection of pancreatic adenocarcinoma. *Am. J. Surg.*, **163**, 68–73
9. Gall, F. (1990) Das duktale Pankreaskarzinom. Langenbecks Arch Chir (Suppl II), **375**, 129–133
10. Cameron, J. L., Pitt, H. A. and Yeo, C. J., *et al.* (1993) One hundred and forty-five consecutive pancreaticoduodenectomies without mortality. *Ann. Surg.*, **217**, 430–438

Johannes Scheele, Richard M. Charnley
and Franz P. Gall
Department of Surgery
University Hospital Erlangen
F. R. G.

### HAS PROPRANOLOL RENDERED SCLEROTHERAPY OBSOLETE FOR POOR RISK ALCOHOLIC CIRRHOTIC PATIENTS?

### ABSTRACT

*Ink, O., Martin, T., Poynard, T., Reville, M., Anciaux, M.-L., Lenoir, C., Marill, J.-L., Labadie, H., Masliah, C., Perrin, D., Chaput, J.-C., Vetter, D., Eugene, C., Lebodic, L., Licht, H. and Etienne, J.-P. (1992) Does elective sclerotherapy improve the efficacy of long-term propranolol for prevention of recurrent bleeding in patients with severe cirrhosis? A prospective multicenter, randomized trial. Hepatology; 16: 912–919*

**We conducted a prospective, multicenter, randomized trial to compare the efficacy of sclerotherapy plus propranolol with that of propranolol alone in the prevention of recurrent gastroesophageal bleeding in severely cirrhotic patients. For 2 yr (1987 to 1988) 131 patients (96% of whom were alcoholic) with Child-Pugh class B or C cirrhosis (56% were class B and 44% were class C) were randomly assigned to one of our two treatment groups after cessation of variceal bleeding, without hemostatic sclerosis, and were observed for at least 2 yr. Treatment observance was good in 89% of cases; alcohol withdrawal was observed in 62% of cases. Sclerotherapy was performed weekly with 1% polidocanol, and variceal obliteration was obtained in 83% of cases, in a mean number of four sessions. The cumulative percentages (expressed as mean $\pm$ S.D.) of recurrent bleeding at 2 yr were 42% $\pm$ 6% for propranolol plus sclerotherapy and 59% $\pm$ 6% for propranolol alone (a nonsignificant difference). Twenty-eight patients from the propranolol group but only 12 patients from the propranolol-plus-sclerotherapy group had recurrent bleeding from esophageal variceal rupture ($p < 0.01$). The total number of blood units per patient with recurrent bleeding was slightly but not significantly more important in the propranolol group (8 $\pm$ 7) than in the propranolol-plus-sclerotherapy group (5 $\pm$ 5; $p = 0.09$). There were no statistical differences in the cumulative survival rate at 2 yr (propranolol plus sclerotherapy, 74% $\pm$ 6% and propranolol alone, 64% $\pm$ 6%) or in the number of patients who died of repeat bleeding (propranolol plus sclerotherapy, 13% $\pm$ 4% and propranolol alone, 17% $\pm$ 5%). Among the surviving patients, cirrhosis**

**improved during the follow-up; Child-Pugh classification was the following at 1 mo: 35% for class A, 50% for class B and 15% for class C and the following at 2 yr: 58% for class A, 38% for class B and 4% for class C. In conclusion, elective sclerotherapy does not significantly decrease the rate of recurrent bleeding or death in severely cirrhotic patients who are treated with propranolol and who mainly abstain from drinking alcohol. (Hepatology 1992; 16:912–919.)**

KEY WORDS: Portal hypertension; bleeding oesophageal varices; sclerotherapy; propranolol; alcoholic cirrhosis.

### PAPER DISCUSSION

This is the first report of a prospective randomized trial comparing sclerotherapy plus propranolol and propranolol alone for prevention of recurrent bleeding in patients with severe alcoholic cirrhosis. They found no beneficial effects of elective sclerotherapy regarding the efficacy of long-term propranolol; the only positive finding was a decreased rate of recurrent episodes of bleeding from oesophageal varices after sclerotherapy[1].

Lebrec *et al.*[2,3] seemed to have been the first to use propranolol for prevention of recurrent bleeding from oesophageal varices; they reported a high rate of bleeding (36%) during a 2 year period. The rates of recurrent bleeding and mortality are comparable with those reported by other workers[4,5]. The mortality may partly be due to liver failure in patients with Child's C liver functions, because of a reduction in portal pressure; the objective of propranolol administration. The decreased rate of variceal bleeding may counter-balance any deterioration in the liver function by decreased portal flow.

Two other forms of prospective randomized trials have been carried out for the evaluation of propranolol and sclerotherapy; propranolol vs. sclerotherapy, and sclerotherapy plus propranolol vs. sclerotherapy. Propranolol seems comparable with sclerotherapy for prevention of variceal bleeding[5,6]. There are trials[7,8] suggesting that propranolol can reduce recurrent gastro-oesophageal bleeding in cirrhotic patients during the course of elective sclerotherapy. In the present trial in which propranolol alone or propranolol plus sclerotherapy was compared, there were no obvious benefits of sclerotherapy in the course of this therapy.

One possible explanation for the failure of any enhancing effect of sclerotherapy in patients treated with propranolol is the technique used for sclerotherapy, as well as the entry criteria and follow-up. The oral administration of propranolol presents no problem and intolerance by the patients is the only untoward event. On the other hand, effective sclerotherapy requires much experience to obtain good results, otherwise, there will be a higher rate of recurrent bleeding from the varices. There are variants of sclerotherapy technique with some showing higher bleeding rates and a high mortality[5,9], albeit lower than those in the study in question[1]. The type of sclerosant, injection intervals, rigid follow-up and endpoint[10] are vital factors to achieve the intended outcome. The high rate of bleeding from varices and the ensuing ulceration in the early stage of sclerotherapy may explain the high mortality rate due to bleeding and liver failure in their study[1]. Bleeding was the result of an incomplete thrombosis not only in the main variceal channels but also in the surrounding small channels. There was a high rate of bleeding episode before variceal obliteration in the sclerotherapy group (56%, 14 out of 25); much higher than in other series[5,11]. At 2 years, there was a high rate of recurrent bleeding (42%) which can be attributed to the low rate of variceal eradication (82%), in the present trial. With appropriate techniques, variceal bleeding can be prevented, as there are no varices to bleed if all varices are eradicated. Using an improved sclerotherapy technique a sufficient volume of 5% ethanolamine oleate (a more potent sclerosant than 1% polidocanol) is injected and the ensuing denuding of the distal oesophageal mucosa prevents recurrence of varices, and the rate of bleeding is greatly diminished and the prognosis is much improved[10]. The technique used for sclerotherapy should be given much more focus when evaluating clinical data, because multicenter trials always raise the problem regarding uniformity of technique; in some institutions the bleeding rate may be much higher than in other clinics.

Ink *et al.*[1] clearly showed that the occurrence of repeat bleeding was a major prognostic factor for prediction of survival during the study period; 50% of the patients had recurrent bleeding episodes (30 of 60)

and only 14% of the patients had no recurrent bleeding episodes (10 of 71).

Patients with oesophageal varices were included in the present trial, and judged as variceal bleeding, because there was no other potential source of bleeding; one third of such patients were included in this trial, as cases of variceal bleeding. Bleeding may occur because of gastritis which is very difficult to identify more than 12 hours later. It may be a reasonable finding that elective sclerotherapy did not reduce the rate of upper gastrointestinal bleeding in patients given propranolol. It may be that elective sclerotherapy showed no benefit for prevention of both esophago-gastric variceal bleeding and non-variceal gastric bleeding, because sclerotherapy may elevate portal pressure following the blockage of large collaterals of portal system and bleeding from the stomach, in the presence of increased portal hypertension may occur[12].

In the present trial[1], alcohol withdrawal was observed only in 62% of the patients, although the number was not given separately for the two groups. There was a high rate of "lost to follow up," perhaps due to alcohol abuse. Abstinence from alcohol is a key factor in the follow-up of patients and in achieving improved clinical results after sclerotherapy. Because additional sessions of sclerotherapy are essential; the number of patients with recurrence of varices, and these requiring additional treatment should be documented during the follow-up period.

Indeed there was a significant difference in the rates of recurrent bleeding due to variceal rupture between the two groups; 28 of the 65 patients in the sclerotherapy plus propranolol vs. 12 of 66 patients in the propranolol alone group. The greater the expertise with sclerotherapy the smaller the number of bleeding episodes will become with a greater decrease in mortality.

It is reasonable that elective sclerotherapy did not produce a benefit in the management of patients on propranolol therapy, unless their varices were at high risk of bleeding; this should be confirmed endoscopically. Even in patients with small non-risky varices, sclerotherapy may induce oesophageal bleeding by creating an ulcer at the injection sites, or by precipitating non-variceal bleeding, such as bleeding from gastritis due to elevation in portal pressure. The current series demonstrates that elective sclerotherapy can benefit patients in the prevention of recurrent short and medium term variceal bleeding, despite a high rate of postinjection bleeding.

Sclerotherapy is useful for controlling acute variceal bleeding, not only because this procedure can be performed along with emergency endoscopy for a diagnosis but also because the control rate is high. Long term results after sclerotherapy will improve with appropriate techniques.

### *References*

1. Ink, O., Martin, T. and Poynard, T., *et al.* (1992) Does elective sclerotherapy improve the efficacy of long-term propranolol for prevention of recurrent bleeding in patients with severe cirrhosis? A prospective multicenter, randomized trial. *Hepatology*, **16**, 912–919.
2. Lebrec, D., Poynard, T., Hillon, P. and Benhamou, J. P. (1981) Propranolol for prevention of recurrent gastrointestinal bleeding in patients with cirrhosis. A controlled study. *New England Journal of Medicine*, **305**, 1371–1374.
3. Lebrec, D., Poynard, T. and Bernuau, J., *et al.* (1984) A randomized controlled study of propranolol for prevention of recurrent gastrointestinal bleeding in patients with cirrhosis; a final report. *Hepatology*, **4**, 355–358.
4. Burroughs, A. K., Jenkins, W. J. and Sherlock, S., *et al.* (1993) Controlled trial of propranolol for prevention of recurrent variceal haemorrhage in patients with cirrhosis. *New England Journal of Medicine*, **309**, 1539–1542.
5. Westaby, D., Polson, R. J. and Gimson, A. E. S., *et al.* A controlled trial of oral propranolol compared with injection sclerotherapy for the long-term management of variceal bleeding. *Hepatology*, **11**, 353–359.
6. Fleig, W. E., Stange, E. F. and Hunecke, R., *et al.* (1987) Prevention of recurrent bleeding in cirrhotics with recent variceal hemorrhage: prospective, randomized comparison of propranolol and sclerotherapy. *Hepatology*, **7**, 355–361.
7. Avgerinos, A., Rekoumis, G. and Klomis, C., *et al.* (1991) Endoscopic sclerotherapy (ES) versus the combination of ES and propranolol for the prevention of recurrent gastrointestinal bleeding in cirrhotics. *Gastroenterology*, **100**, A717.
8. Lundell, L., Leth, R. and Lind, T., *et al.* (1990) Evaluation of propranolol for prevention of recurrent bleeding from esophageal varices between sclerotherapy sessions. *Acta Chirurugica Scandinavia.*, **156**, 711–715.
9. Stiegmann, G. V., Goff, J. S. and Michalezt-Onody, P. A., *et al.* (1992) Endoscopic sclerotherapy as compared with endoscopic ligation for bleeding esophageal varices. *New England Journal of Medicine*, **326**, 1527–1532.
10. Kitano, S., Koyanagi, N. and Iso, Y., *et al.* (1987) Prevention of recurrence of esophageal varices after endoscopic injection sclerotherapy with ethanolamine oleate. *Hepatology*, **7**, 810–815.
11. Kitano, S., Iso, Y. and Hashizume, M., *et al.* (1992) Sclerotherapy versus esophageal transection versus splenorenal shunt for the clinical management of esophageal varices in the patients with Child's A and B liver function. A prospective randomized trial. *Hepatology*, **15**, 63–68.
12. Higashi, H., Kitano, S. and Hashizume, M., *et al.* (1991) Gastric bleeding after endoscopic injection sclerotherapy may be fatal. *International Surgery*, **76**, 214–217.

Seigo Kitano, MD
Associate Professor
Department of Surgery I
Oita Medical University
Oita 879-55
Japan

